# Zn(II) and Cu(II) adsorption and retention onto iron oxyhydroxide nanoparticles: effects of particle aggregation and salinity

**DOI:** 10.1186/1467-4866-15-6

**Published:** 2014-05-03

**Authors:** Rebecca B Chesne, Christopher S Kim

**Affiliations:** 1 School of Earth and Environmental Sciences, Schmid College of Science & Technology, Chapman University, Orange, CA 92866, USA

**Keywords:** Metal, Adsorption, Retention, Nanoparticles, Aggregation, Salinity, EXAFS

## Abstract

**Background:**

Iron oxyhydroxides are commonly found in natural aqueous systems as nanoscale particles, where they can act as effective sorbents for dissolved metals due to their natural surface reactivity, small size and high surface area. These properties make nanoscale iron oxyhydroxides a relevant option for the remediation of water supplies contaminated with dissolved metals. However, natural geochemical processes, such as changes in ionic strength, pH, and temperature, can cause these particles to aggregate, thus affecting their sorption capabilities and remediation potential. Other environmental parameters such as increasing salinity may also impact metal retention, e.g. when particles are transported from freshwater to seawater.

**Results:**

After using synthetic iron oxyhydroxide nanoparticles and nanoparticle aggregates in batch Zn(II) adsorption experiments, the addition of increasing concentrations of chloride (from 0.1 M to 0.6 M) appears to initially reduce Zn(II) retention, likely due to the desorption of outer-sphere zinc surface complexes and subsequent formation of aqueous Zn-Cl complexes, before then promoting Zn(II) retention, possibly through the formation of ternary surface complexes (supported by EXAFS spectroscopy) which stabilize zinc on the surface of the nanoparticles/aggregates. In batch Cu(II) adsorption experiments, Cu(II) retention reaches a maximum at 0.4 M chloride. Copper-chloride surface complexes are not indicated by EXAFS spectroscopy, but there is an increase in the formation of stable aqueous copper-chloride complexes as chloride concentration rises (with CuCl^+^ becoming dominant in solution at ~0.5 M chloride) that would potentially inhibit further sorption or encourage desorption. Instead, the presence of bidentate edge-sharing and monodentate corner-sharing complexes is supported by EXAFS spectroscopy. Increasing chloride concentration has more of an impact on zinc retention than the mechanism of nanoparticle aggregation, whereas aggregation condition is a stronger determinant of copper retention.

**Conclusions:**

Based on these model uptake/retention studies, iron oxyhydroxide nanoparticles show potential as a strategy to remediate zinc-contaminated waters that migrate towards the ocean. Copper retention, in contrast, appears to be optimized at an intermediate salinity consistent with brackish water, and therefore may release considerable fractions of retained copper at higher (e.g. seawater) salinity levels.

## Background

Anthropogenic activities such as metal ore mining are known to cause metal contamination of the surrounding environment. Precipitation events and regular seepage/flow in these areas mobilize acidic water and dissolved metals, contaminating nearby water sources [1,2]. While the harmful effects of some metals such as mercury and lead are well known, metals that are common dietary requirements such as zinc and copper can also cause health problems upon elevated exposure. For example, excess copper intake can induce neurological and psychological problems, while elevated zinc can impair the absorption of other ions such as copper and iron and cause corrosive damage to soft tissues [3]. Metal contamination can also disrupt the ecosystems in proximity of these water supplies; for example, in plants excess zinc is found to inhibit many metabolic functions while elevated copper levels induce injury and oxidative stress, also disturbing metabolism and macromolecular activity [4].

Iron oxyhydroxides, specifically goethite (α-FeOOH) and ferrihydrite ((Fe)_2_O_3_•0.5 H_2_O), form readily in acid mine drainage environments. In these systems, iron oxyhydroxides form when pyrite reacts upon exposure to oxygen and water [5], producing large volumes of acid that further facilitate the dissolution of sulfides and the mobilization of trace metals. Iron oxyhydroxides occur at the nanoscale in natural environments [6-8] and are inherently effective sorbents for dissolved metals because of their small size, high surface area, and natural surface reactivity [9-13]. Iron oxyhydroxides are also considered to be the predominant reactive mineral phase in lake and marine sediments [8], where they can play a significant role in natural attenuation processes.

Typically, iron oxyhydroxide nanoparticles aggregate rapidly [14,15] upon exposure to natural geochemical conditions based on ionic strength, pH, and temperature, although the mechanisms of aggregation vary for each of these parameters. Increasing pH decreases the particles’ surface charge density as pH approaches the point of zero charge, which for iron oxyhydroxides is between pH 7.0 and 9.0 [16]. As the surface charge decreases, electrostatic repulsion between particles is reduced, allowing them to move closer together and aggregate [17-19]. Alternatively, increasing ionic strength decreases the electrical double layer thickness needed to offset the particles’ surface charge [20-22], enabling particle aggregation. Lastly, increasing temperature increases thermal motion of the nanoparticles, causing them to collide more often and with more force, resulting in aggregation [23].

These differences in aggregation mechanism lead to the formation of morphologically distinct aggregates. For example, increased pH and ionic strength induce rapid aggregation, leading to the formation of disordered fractal aggregates [23,24], while increased temperature allows aggregation to occur more slowly, resulting in the formation of more ordered and oriented aggregates [23]. The differing morphologies of nanoparticle aggregates exposed to varying geochemical conditions can affect their metal retention properties by reducing available reactive surface area, inhibiting (or creating) access to aggregate interpore spaces, and by altering the proportions of different surface binding sites [25-27].

The salinity of the aqueous environment can also affect metal adsorption/retention capabilities. Although it has been shown that the presence of chloride typically inhibits metal uptake onto iron oxyhydroxides through the formation of stable metal-chloride aqueous species [12,20,28], less is known about the retention behavior of metals that were initially sorbed to nanoparticles or their aggregates in a freshwater environment before then being exposed to increasing salinity. Simulating the transition from freshwater streams and rivers (represented by 0 M chloride) to brackish (mixed) waters of bays and estuaries (0.1-0.4 M chloride) to marine waters (0.6 M chloride) will enable a better understanding of how increasing salinity levels affect metal retention to the nanoparticles. The effect of nanoparticle aggregation, representing the conditions that the particles were exposed to prior to metal uptake, is an additional determinant potentially controlling metal adsorption and retention.

Previous studies indicate that different aggregation mechanisms produce nanoparticle aggregates with varying metal retention capabilities [23,29]. This study will investigate the effect of chloride concentration and a subsequent lowering of pH on Zn(II) and Cu(II) retention to unaggregated iron oxyhydroxide nanoparticles as well as those aggregated under conditions of elevated ionic strength, pH, and temperature. Our hypothesis is that introducing metal-sorbed iron oxyhydroxide nanoparticles or nanoparticle aggregates to an increasingly saline environment, as in the transition from freshwater to seawater, will systematically reduce Zn(II) and Cu(II) retention onto nanoparticle surfaces. The expectation is also that more aggregated particles will retain less metal initially but will also be less influenced by increasing salinity, thus exhibiting greater retention than unaggregated or less aggregated nanoparticles.

## Experimental methods

### Iron oxyhydroxide nanoparticle synthesis

Iron oxyhydroxide nanoparticles were prepared using a flash microwave synthesis technique described by Guyodo et al. [30]. Equal volumes of 0.20 M Fe(NO_3_)_2_ and 0.25 M NaHCO_3_ solutions were prepared before adding the NaHCO_3_ to the Fe(NO_3_)_2_ dropwise through a 0.20 μm syringe filter. Once mixing was complete, the solution was agitated on a shaking table and vented periodically until the newly formed CO_2_ in solution was released (approximately 5 minutes total). After degassing, the solution was heated in a conventional microwave at high intensity for approximately 3.5 minutes in 30-second intervals just until the onset of boiling to induce nucleation. To halt nucleation, the resulting nanoparticle suspension was immediately placed in an ice bath until it had returned to room temperature. The suspension was transferred into 1000 MWCO dialysis tubing and allowed to equilibrate in deionized water which was replaced 3 times/day until the pH and conductivity had stabilized at 5.0 and 1.5 μS/cm, respectively (~5 days). Following equilibration, the resulting nanoparticle suspension (final solids concentration: 6.7 g/L) was refrigerated at 4°C in sealed HDPE bottles until used in aggregation and/or batch uptake experiments.

### Nanoparticle aggregation

Separate aliquots of the initial nanoparticle suspension were exposed to varying pH, ionic strength, and temperature conditions in order to induce aggregation (Table 1). To aggregate the particle suspension under increased pH and ionic strength conditions, it was first transferred into lengths of 1000 MWCO dialysis tubing and placed into a control solution (0.001 M NaNO_3_ and pH 5.0) for 3 days to allow equilibration. The particle suspensions, still in the same dialysis tubing sections, were then transferred into separate solutions of their respective aggregation conditions (Table 1). The aggregation solutions were replaced daily. After 5 days, the particle suspensions were returned to control solution to allow re-equilibration for 3 days, with the control solution also replaced daily. The particle suspensions were then refrigerated at 4°C until they were characterized and used in uptake experiments.

**Table 1:** Experimental aggregation conditions listing ionic strength, pH, and temperature

***Aggregation condition***	**NaNO**_**3 **_**concentration**	**pH**	**Temperature**
**Control**	0.001 M	5.0	RT (~20°C)
**pH 8**	0.001 M	8.0	RT (~20°C)
**pH 10**	0.001 M	10.0	RT (~20°C)
**0.1 M**	0.1 M	5.0	RT (~20°C)
**1.0 M**	1.0 M	5.0	RT (~20°C)
**25°C**	0.001 M	5.0	25°C
**50°C**	0.001 M	5.0	50°C
**75°C**	0.001 M	5.0	75°C

To aggregate the particle suspensions at elevated temperatures, aliquots of the suspension were placed into tightly-capped HDPE bottles. The bottles were placed in ovens at their respective temperatures (Table 1) for 4 days. After 4 days of heating, the particle suspensions were refrigerated at 4°C until their use in further experiments.

### Characterization of nanoparticle aggregates

#### X-ray diffraction

Powder X-ray diffraction patterns were collected on control (unaggregated) nanoparticles as well as those aggregated at 0.1 M ionic strength, 1.0 M ionic strength, pH 8.0, and pH 10.0 to assess the crystallinity and mineral phase of the samples. Samples were air-dried in 50 mL Falcon centrifuge tubes and ground with an agate mortar and pestle prior to loading as a thin film between two layers of Scotch tape. X-ray diffraction patterns were collected at the Stanford Synchrotron Radiation Lightsource (SSRL) on beamline 11-3 at an energy of 12735 eV using a Si(311) crystal monochromator calibrated with a powdered LaB_6_ standard. Data was collected using a Mar345 CCD detector for 90 seconds for all samples except for the pH 8.0 sample, for which data was collected for 30 seconds and scaled accordingly. The resulting patterns were analyzed with the program fit2D [31] and background-subtracted using the program XRD-BS [32].

#### Dynamic light scattering

Dynamic light scattering analysis was conducted on the control nanoparticles and all aggregates to examine and compare their hydrodynamic diameters. Aliquots of the particle suspensions were diluted 10x with deionized water for optimal particle detection and placed into cuvettes, which were loaded directly into a Malvern Zetasizer Nano S dynamic light scattering unit. The samples were agitated directly in their cuvettes multiple times with a 1000 μL pipet immediately before analysis in an effort to minimize the effect of particle settling. Each sample was analyzed between 2–6 times with 70 cumulative measurements collected per trial, with the entire analysis taking approximately 1 minute.

### Geochemical modeling

Geochemical modeling was performed using thermodynamic equilibrium constants for Zn(II) [33] and Cu(II) [34], producing speciation diagrams representing the parameters of the experiment. The concentrations of aqueous Cu(II) and Zn(II) used were based upon the average percent uptake following the adsorption phases of the macroscopic experiments (0.131 mM Cu(II) and 0.046 mM Zn(II) left in solution). The fraction of each metal-chloride compound was calculated as a function of environmental chloride concentration using the relevant thermodynamic equilibrium equations and β values, finding the concentration of each compound by factoring the solubility product of sodium chloride into the equilibrium.

### Metal adsorption/desorption studies

Prior to initiating metal uptake, the iron concentrations of the unaggregated control suspension and each aggregate nanoparticle suspension were measured as a proxy for nanoparticle concentration with a Thermo Scientific SOLAAR M Series atomic absorption spectrometer. The particle suspension was diluted in a 1:216 ratio in water acidified with ultrapure nitric acid (pH <2.0, 0.03 M) in order for the sample iron concentrations to fall within the optimal (linear) detection range of the AA spectrometer. Iron concentrations of the aliquots of aggregate suspensions ranged from 3365 to 3654 ppm. This measurement captures sampling variability as a result of particle aggregation, allowing for normalization based on iron concentration in each aliquot of aggregate suspension added to the experimental setup. The amount of each suspension added to the experimental setups was correspondingly adjusted in order to deliver a consistent quantity of nanoparticles to each reaction vessel, minimizing the likelihood that differences in uptake and desorption behavior between the various aggregates were a function of varying nanoparticle concentration.

Once normalized, iron oxyhydroxide nanoparticle suspensions were exposed to a 5 mM metal (Cu(II) or Zn(II)) stock solution, adding appropriate volumes of DI water, a stock 0.1 M NaNO_3_ solution, and 0.1 M NaOH and/or 0.1 M HNO_3_ to achieve a final target volume of 150 mL, metal concentration of 0.5 mM, and nanoparticle solids concentration of 0.17 g/L while also maintaining ionic strength and pH at control conditions of 0.001 M and pH 5.0. All experiments were conducted in triplicate. After introducing the particle suspensions to the dissolved metal solution, the pH was raised with 20 μL aliquots of 0.1 M NaOH to 6.0 ± 0.1 for Cu(II) samples and 7.0 ± 0.1 for Zn(II) samples to allow for maximum metal uptake. These pH values were determined by preliminary pH-based uptake experiments and calculations as well as those of other investigators [16]. The samples were then sealed in HDPE bottles and placed on a rotating table for 24 hours.

After the adsorption step, the samples were split into three separate 50 mL aliquots. One aliquot was immediately analyzed to assess the initial extent of metal uptake, referred to from this point forward as the “adsorbed” sample. Appropriate amounts of solid NaCl were added to the remaining aliquots to raise the salinity to either 0.1 M, 0.4 M, or 0.6 M (“salt added” samples). For the “salt added” samples, the target salinity level was maintained for 24 hours. For the remaining aliquot, after the salinity increase the pH was lowered to 5.0 ± 0.1 using 20 μL aliquots of 0.1 M HNO_3_ (“pH dropped” samples) and the suspension agitated for 24 hours. While kinetics were not monitored in this study, the 24-hour exposure periods were determined based on our previous ion selective electrode lab studies which showed that adsorption and desorption are each complete after one hour. Exposure periods of 24 hours were used to better simulate environmental conditions and to ensure thorough adsorption and desorption.

Following exposure, samples were centrifuged at 3000 rpm in 50 mL Falcon tubes for 10 minutes. The resulting supernatant was decanted, filtered using 0.20 μm syringe filters, diluted 10x in acidified (pH < 2.0) DI water, and analyzed for either Zn(II) or Cu(II) using AA spectrometry. The metal concentration obtained from AA analysis was used to calculate the degree of metal uptake/retention to the substrates following each of the experimental steps, assuming minimal uptake of dissolved metal to the vessel walls.

### Extended X-ray absorption fine structure (EXAFS) spectroscopic analysis

Selected solid samples from the Zn(II) and Cu(II) macroscopic experiments were collected for EXAFS analysis. The solids were spread onto Whatman filter paper to remove excess water and loaded into Teflon sample holders with Kapton tape as moist pastes for analysis.

All Zn(II) K-edge EXAFS data were collected on the moist pastes at SSRL on beamline 4–3 in fluorescence mode at room temperature using a 13-element high-throughput germanium detector. Cu(II) K-edge EXAFS data were collected at SSRL on beamline 11–2 using a 100-element high-throughput germanium detector in fluorescence mode. The fluorescence method is advantageous for lower concentration samples [35,36]. Aluminum filters were used to reduce Fe K-edge fluorescence and zinc and copper metal foils were used as calibrants.

The resulting spectra were analyzed using the SIXPack data processing software version 1.01 [37]. Deadtime corrections were performed on each scan in order to accommodate for loss of signal upon saturation of the detector channels prior to being averaged together. Those averages were converted to k-space with a k^3^ weighting and were Fourier transformed. All copper average files (excluding the spectra from the pH 10 aggregates at 0.4 M chloride) required minor deglitching due to monochromator imperfections. Both Zn and Cu EXAFS spectra were fit over a k-range of 3.0-12.0 Å using model scattering paths which were generated in SIXPack using Feff6l [38].

## Results and discussion

### Nanoparticle characterization

#### X-ray diffraction

**Figure 1:**
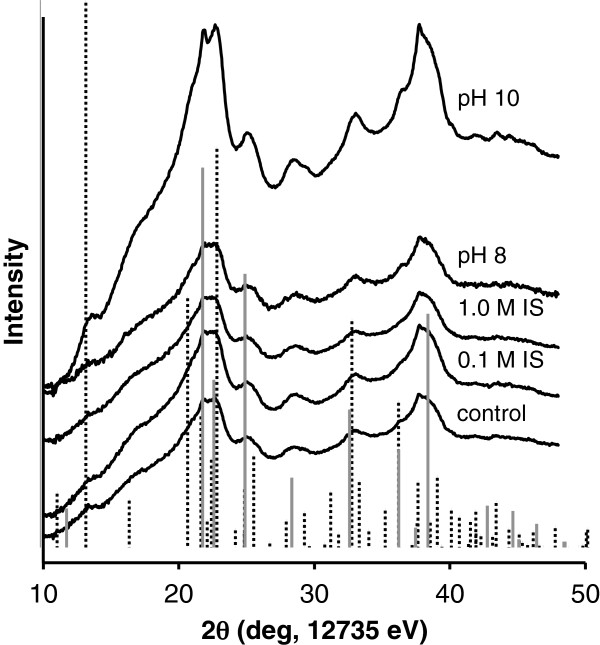
**Powder X-ray diffraction patterns of the control, ionic strength and pH aggregated particles.** Ferrihydrite (solid gray lines [18]) and goethite (dashed lines, No. 29–0713) PDFs are included for comparison.

Generally, the XRD patterns obtained from the aggregated particles are similar to those of the unaggregated control particles (Figure 1), suggesting that exposing the nanoparticle suspension to aggregation conditions does not significantly alter the mineral phase or degree of crystallinity. The scattering pattern of the pH 10-aggregated particles shows higher peak intensities consistent with larger aggregates. X-ray diffraction analysis was not performed on temperature-aggregated particles in this study; however, based on our previous studies [29], aging these same iron oxyhydroxide nanoparticles at 78°C caused a gradual transformation from ferrihydrite to goethite over a 7-day duration.

Comparison of the collected diffraction patterns to PDF standards of 6-line ferrihydrite and goethite (Figure 1) indicates that the nanoparticle suspensions are primarily comprised of 6-line ferrihydrite. However, there are a few specific peaks present that are unique to goethite. The particles therefore appear to be nanoparticulate ferrihydrite, but some proportion of the sample is goethite or has some degree of goethite-like structure.

#### Dynamic light scattering

**Figure 2:**
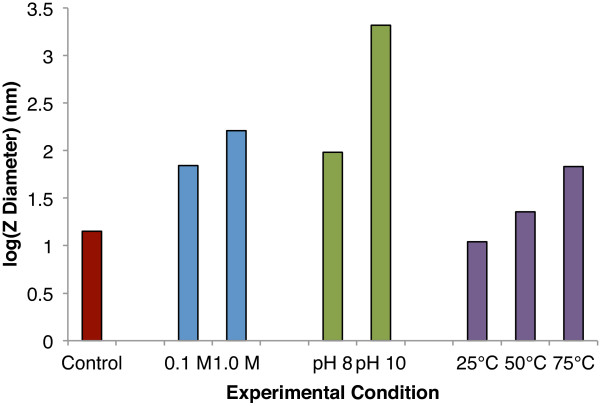
**Hydrodynamic diameters of control and aggregated nanoparticles measured using dynamic light scattering analysis.** Results are shown on a log scale.

Analysis of the nanoparticle aggregates with dynamic light scattering demonstrates that as the nanoparticles are exposed to elevated pH, ionic strength, or temperature, their z-diameter increases, indicating the formation of larger aggregates (Figure 2). This confirms that aggregation is induced by these parameters and further verifies that returning the aggregates to control solution following exposure to aggregation conditions is not sufficient to provoke significant reversal of the aggregation.

The results also suggest that elevated pH or ionic strength conditions produce larger aggregates than elevated temperature over the timeframes investigated and is indicative of different aggregation mechanisms and rates. When the nanoparticles are placed in environments with elevated temperature, they aggregate slowly (and often in an oriented manner), forming relatively compact, ordered aggregates. In contrast, nanoparticles exposed to elevated pH or ionic strength aggregate more rapidly, forming disordered aggregates with substantial interstitial water and resulting in a larger aggregate diameter [23]. Dynamic light scattering results are consistent with X-ray diffraction results, both of which suggest that higher pH, ionic strength, and temperature induce greater degrees of aggregation, with particles aggregated at pH 10 being the largest. Earlier small and wide-angle X-ray scattering studies [23] also support these findings of increased aggregation with elevated pH, ionic strength, and temperature.

### Zinc sorption

#### Macroscopic results

**Figure 3:**
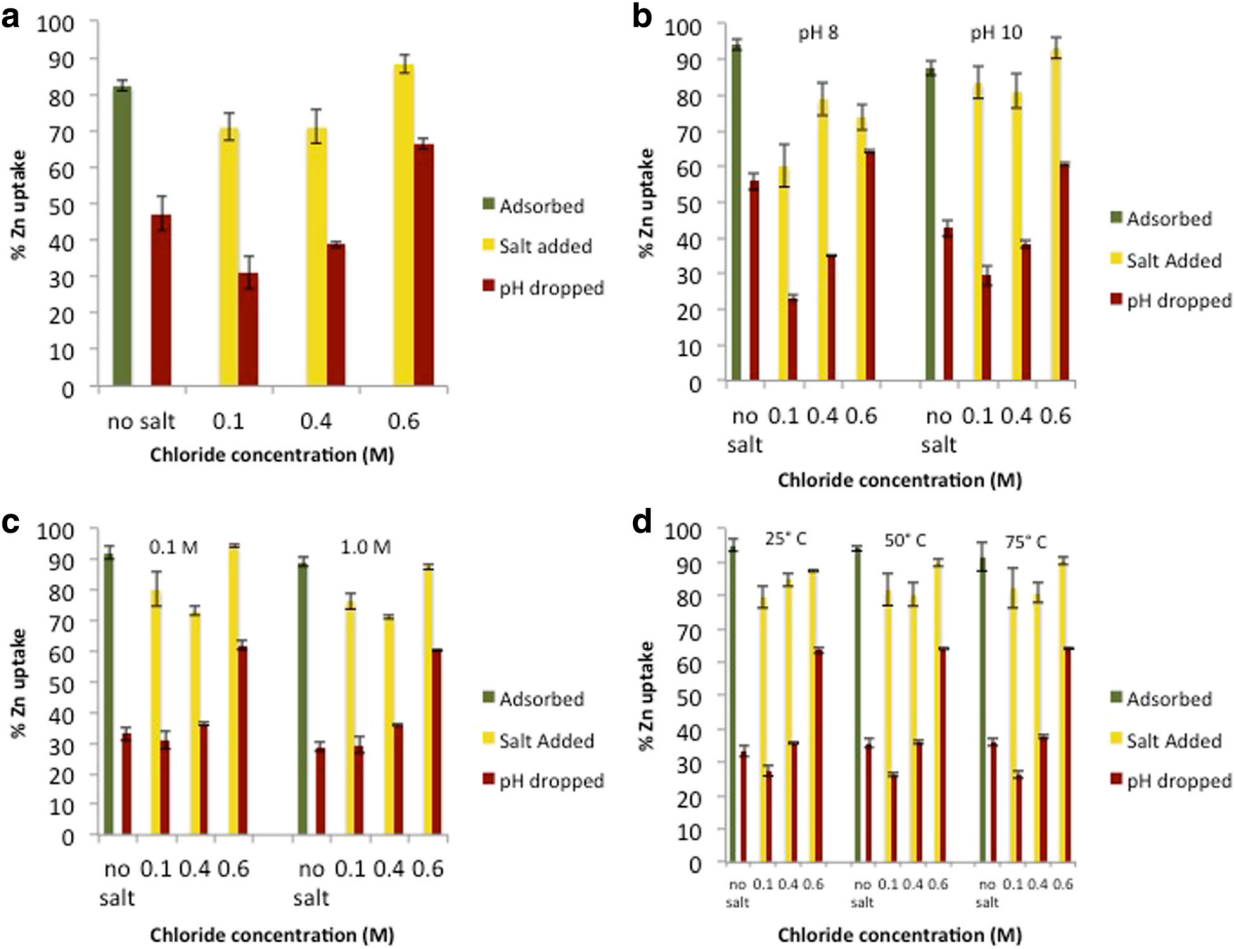
**Macroscopic zinc uptake/retention data for a) control, b) pH-aggregated, c) ionic strength-aggregated, and d) temperature-aggregated nanoparticles.** “Adsorbed” samples (green columns) represent pH 6.0 ± 0.1, 0.1 M NaNO_3_, “salt added” samples (yellow columns) represent pH 6.0 ± 0.1 and 0.1-0.6 M NaCl, and “pH dropped” samples (red columns) represent pH 5.0 ± 0.1, 0.1 M NaNO_3_, 0.1-0.6 M NaCl.

Raising the pH to 7.0 ± 0.1 in the adsorption phase caused 85-95% of the zinc in solution to sorb to the particle surfaces (“adsorbed” samples, Figure 3a-d). On average, the initial addition of chloride caused minor desorption to occur (0.1 M “salt added” samples) relative to the substantial decrease in zinc retained when the pH was lowered in the presence of chloride (“pH dropped” samples). In both the “salt added” and “pH dropped” samples, a general increase is seen in the percent zinc retained as chloride concentration increases, following a slight drop in percent zinc retained upon the addition of 0.1 M NaCl, with the trend appearing much more consistently in the “pH dropped samples” (Figure 3a-d). These results suggest that increasing chloride concentration stabilizes the zinc that is sorbed to the particle surfaces, causing more zinc to be retained.

The initial decline in zinc retention after lowering pH between the salt-free and 0.1 M salinity trials (Figure 3a-d) suggests that it may be more thermodynamically favorable for free or weakly-bound zinc, such as zinc in outer-sphere sorption complexes, to form stable aqueous zinc-chloride species over surface sorption complexes within this salinity range. As the chloride concentration rises, however, more chloride is available to react with zinc sorbed through inner-sphere mechanisms. It is possible that chloride modifies the type of surface complex formed between the zinc and the nanoparticle, stabilizing the Zn-surface bond.

**Figure 4:**
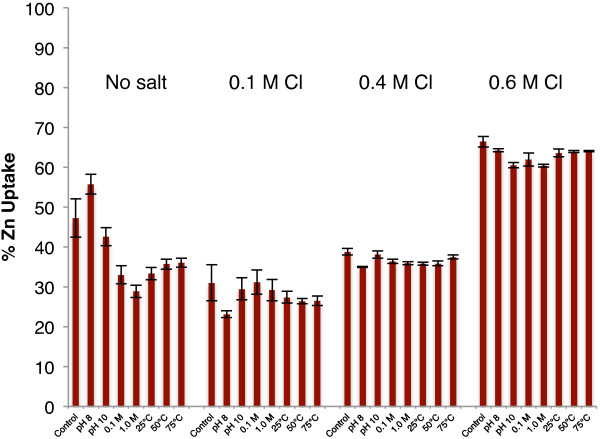
**Macroscopic zinc retention data for all samples at all salinities following exposure to NaCl for 24 hours at pH 5.0 ± 0.1.** Salinity increases from left to right as labeled.

Comparing only the retention data from the “pH dropped” samples highlights the initial decline in retention from the salt-free to 0.1 M Cl^−^ conditions and the progressive increase in Zn(II) retention as the salinity is further increased to 0.6 M (Figure 4). Upon introducing a saline environment, the differences in retention between aggregates decreased (as assessed by the standard errors within each salinity category shown in Table 2, calculated using results from all samples), indicating that the effect of increased environmental chloride concentration is stronger than the effect of the aggregation method on the retained fraction.

**Table 2:** Average percent Zn(II) uptake and standard errors for “pH dropped” samples

	**Average (% uptake)**	**Standard error**
**No salt**	39.1%	3.14%
**0.1 M Cl**^**−**^	28.1%	0.96%
**0.4 M Cl**^**−**^	36.7%	0.47%
**0.6 M Cl**^**−**^	63.2%	0.73%

#### Geochemical modeling

**Figure 5:**
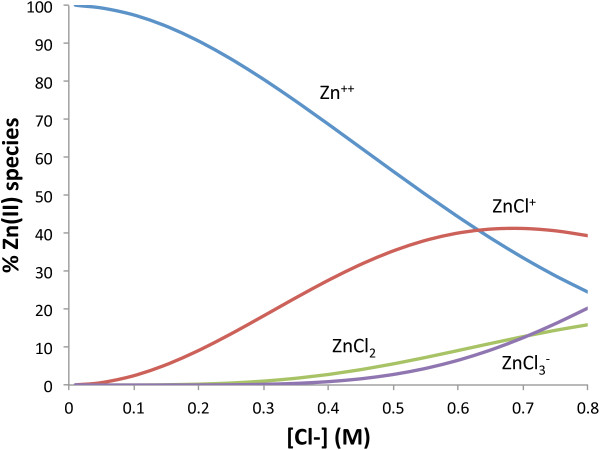
Speciation diagram of Zn(II) chloride species at a range of chloride concentrations and a Zn(II) concentration of 0.046 mM at pH 5.0.

As the environmental chloride concentration increases, the fraction of Zn^2+^ in solution decreases as aqueous zinc-chloride complexes are formed (Figure 5). ZnCl^+^ forms the fastest, overtaking Zn^2+^ as the dominant aqueous zinc species at ~0.65 M chloride, although ZnCl_2_ and ZnCl_3_^−^ are also present in smaller proportions (ZnCl_4_^2−^ is not shown because its corresponding β value was not provided). This experiment only tested zinc retention up to 0.6 M chloride, but previous macroscopic studies conducted in our lab suggest that zinc retention plateaus at chloride concentrations between 0.6 M and 1.2 M, which could correlate with the dominant zinc species becoming ZnCl^+^ at 0.65 M chloride.

#### Spectroscopic results

**Figure 6:**
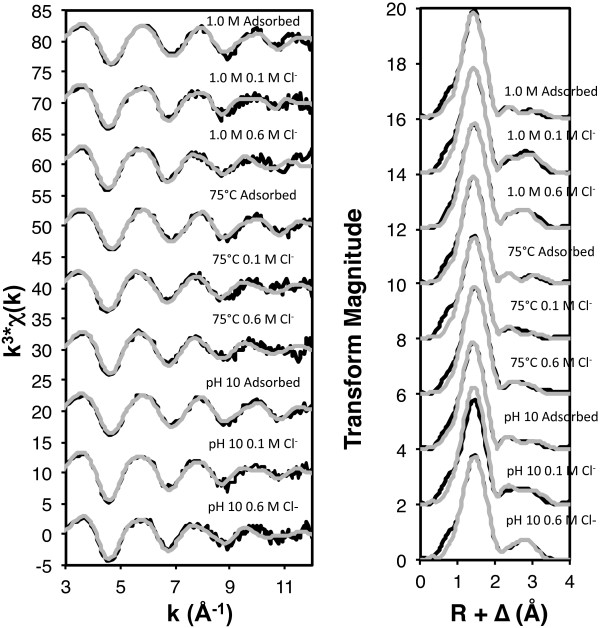
**Zinc K-edge EXAFS spectra and Fourier transforms (black) with overlain fits (grey) for “adsorbed” and “pH dropped” states (0.1 M Cl**^**− **^**and 0.6 M Cl**^**−**^**) of samples aggregated at 1.0 M, 75°C, and pH 10.**

Subtle spectral differences can be observed between the adsorbed samples and those whose pH levels were lowered in the presence of chloride (Figure 6). These differences are most apparent in the Fourier transforms, in which the second–neighbor features appear to merge together as chloride concentration increases. The EXAFS fitting results of the first-neighbor feature show an average coordination number of 3.6 (range: 3.6-3.7) (Table 3) for the Zn-O shell in the adsorbed samples, indicating that in the adsorption phase, sorbed zinc is initially binding to the nanoparticle surfaces in a dominantly tetrahedral coordination environment. These results, in conjunction with previous studies and fitted Zn-O bond distances of 1.99 ± 0.01 Å, suggest that zinc is forming bidentate corner-sharing complexes with the iron octahedra that comprise the nanoparticle surfaces [29,39], consistent with zinc binding to ferrihydrite [39-42]. The increase in the Zn-O coordination number of the “pH dropped” samples (average: 5.4, range: 5.2-6.1) compared to that of the “adsorbed” samples (average: 3.6) also indicates a shift from the zinc being bound on less ordered, tetrahedrally-coordinated sites to more ordered, octahedrally-coordinated sites.

**Table 3:** **Results from K-edge EXAFS fitting of Zn(II) samples (see Figure**6**for corresponding EXAFS spectra and Fourier transforms)**

		**Zn-O**		**Zn-Cl**		**Zn-Fe 1**		**Zn-Fe 2**		
**Aggregation condition**	**Sorption step**	**CN**	**R (Å)**	**CN**	**R (Å)**	**CN**	**R (Å)**	**CN**	**R (Å)**	**R-Factor**
**1.0 M**	**Adsorbed**	3.6 ± 0.5	1.98 ± 0.01			0.9 ± 0.4	3.18 ± 0.05	0.5 ± 0.3	3.39 ± 0.05	0.0027
**1.0 M**	**0.1 M Cl**^**−**^	5.2 ± 1.0	2.01 ± 0.01	0.8 ± 0.4	2.99 ± 0.06			1.4 ± 0.5	3.46 ± 0.04	0.0045
**1.0 M**	**0.6 M Cl**^**−**^	5.4 ± 0.8	2.02 ± 0.01	1.8 ± 0.5	2.99 ± 0.05	1.6 ± 0.5	3.05 ± 0.05			0.0024
**75°C**	**Adsorbed**	3.6 ± 0.4	1.98 ± 0.01			0.9 ± 0.3	3.16 ± 0.03	0.4 ± 0.2	3.42 ± 0.04	0.0014
**75°C**	**0.1 M Cl**^**−**^	5.2 ± 0.9	2.03 ± 0.01	0.7 ± 0.3	2.95 ± 0.05			0.5 ± 0.4	3.45 ± 0.07	0.0039
**75°C**	**0.6 M Cl**^**−**^	5.2 ± 0.8	2.04 ± 0.01	1.3 ± 0.5	3.01 ± 0.06	0.9 ± 0.5	3.07 ± 0.07			0.0032
**pH 10**	**Adsorbed**	3.7 ± 0.3	1.98 ± 0.01			1.0 ± 0.3	3.18 ± 0.03	0.6 ± 0.3	3.39 ± 0.05	0.0012
**pH 10**	**0.1 M Cl**^**−**^	6.1 ± 1.2	2.02 ± 0.02	1.0 ± 0.4	2.97 ± 0.05			1.1 ± 0.5	3.45 ± 0.04	0.0041
**pH 10**	**0.6 M Cl**^**−**^	5.5 ± 0.6	2.03 ± 0.01	1.8 ± 0.4	2.99 ± 0.04	1.7 ± 0.4	3.05 ± 0.03			0.0015

Included are coordination numbers (CN), interatomic distances (R), and goodness of fit values (R-factor). Debye-Waller values were allowed to float for first-shell fits (average: 0.004 Å^2^) and were fixed at 0.01 Å^2^ for subsequent shells.

The adsorbed samples were best fit with two Zn-Fe shells, the first with an average coordination number of 0.9 (range: 0.9-1.0) and bond length of 3.17 Å (range: 3.16-3.18 Å), and the second with an average coordination number of 1.1 (range: 1.0-1.1) and bond length of 3.42 Å (range: 3.41-3.43 Å) (Table 3). These results generally correlate with Juillot et al. [39], who reported bond lengths of 3.47 Å for zinc bonded to ferrihydrite and 3.07 Å and 3.26 Å for goethite. The long bonds present in the ferrihydrite samples indicate the presence of bidentate corner-sharing complexes [39,43], while the shorter Zn-Fe bonds in the goethite samples correspond to bidentate (3.26 Å) and tridentate (3.07 Å) face-sharing complexes [39,44]. The presence of both ferrihydrite and goethite binding sites is consistent with the X-ray diffraction results, which indicate that the nanoparticle suspensions exhibit features consistent with both ferrihydrite and goethite (Figure 1). The adsorbed samples were best fit with bond lengths correlating with past studies on both goethite and ferrihydrite, and indicate contributions from bidentate edge-sharing (3.17 Å) and bidentate corner-sharing (3.42 Å) complexes (Table 3).

As the environmental chloride concentration is increased, there are consistent changes to the Zn-Fe fits. At 0.1 M chloride, the best fits resulted in an average Zn-Fe coordination number of 1.0 (range: 0.5-1.4) and bond length of 3.46 Å (range: 3.45-3.46 Å) (Table 3), which most accurately indicates bidentate corner-sharing complexes, as seen in previous ferrihydrite studies [39,43]. With increasing chloride concentrations, the observed Zn-Fe bond lengths shorten (average: 3.06 Å, range: 3.05-3.07 Å) and coordination numbers increase (average: 1.4, range: 0.9-1.7) (Table 3), corresponding to the presence of tridentate face-sharing complexes [39,44].

The identification of Zn-Cl neighbors in the EXAFS fitting results indicates that zinc is on average bound to more chloride ions as the chloride concentration of the experimental system increases, with the coordination number approaching a value of 2. The Zn-Cl coordination numbers indicate that octahedrally-coordinated zinc has up to 2 chloride ligands, which would reduce surface charge repulsion and sorb as an uncharged ternary surface complex. An average Zn-Cl interatomic distance of 2.98 Å (2.95-2.99 Å) is consistent with a direct Zn-Cl bond [45], supporting the conclusion that zinc and chloride are binding directly to each other (Table 3). The results from the macroscopic experiments and EXAFS analysis suggest that the octahedral surface complexes bound to more ordered binding sites showing evidence of a direct Zn-Cl bond and tridentate face-sharing complexes are more stable, because the conditions under which these complexes are formed showed higher percentages of retained zinc.

### Copper sorption

#### Macroscopic results

**Figure 7:**
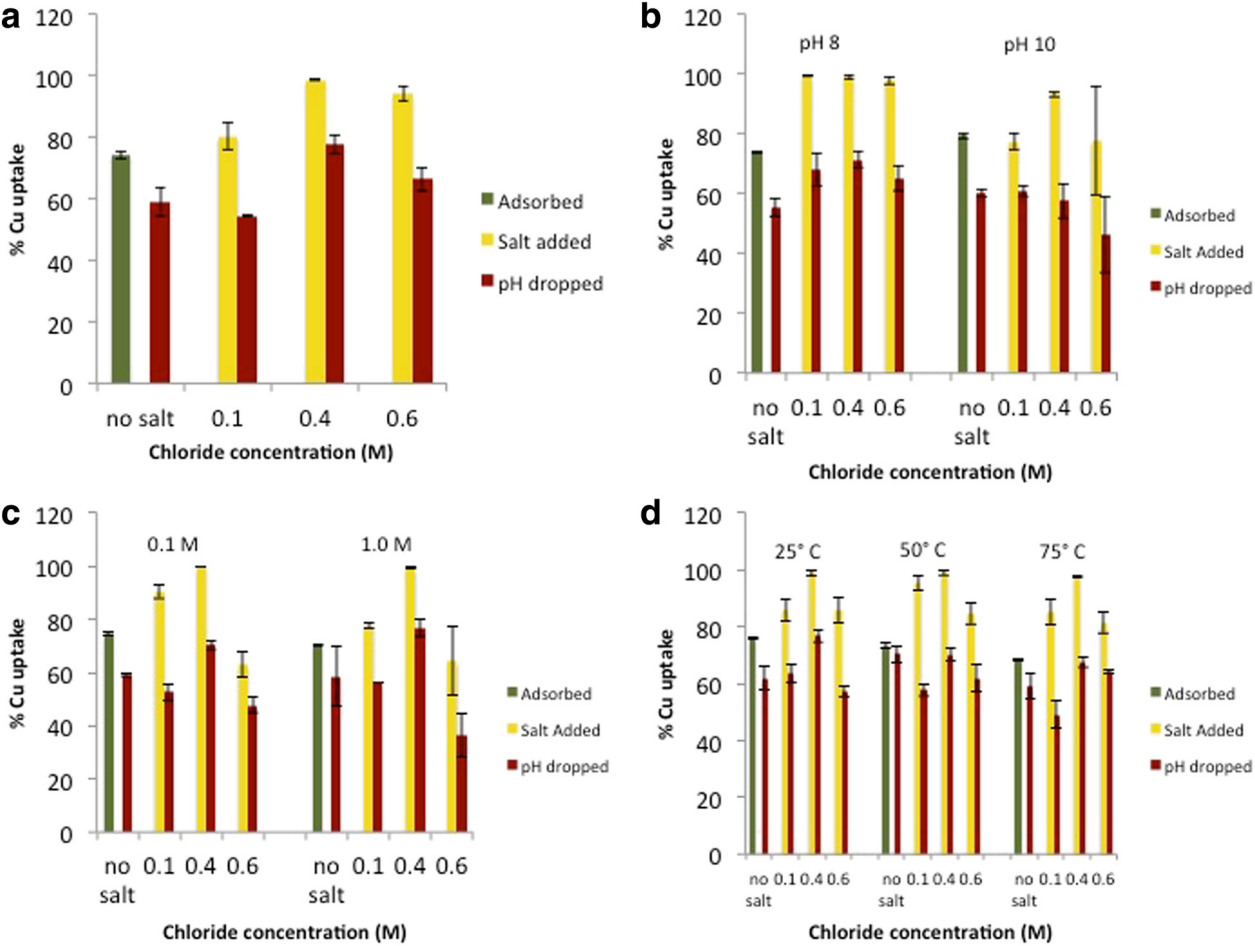
**Macroscopic copper uptake/retention data for a) control, b) pH-aggregated, c) ionic strength-aggregated, and d) temperature-aggregated nanoparticles.** “Adsorbed” samples (green columns) represent pH 7.0 ± 0.1, 0.1 M NaNO_3_, “salt added” samples (yellow columns) represent pH 7.0 ± 0.1 and 0.1-0.6 M NaCl, and “pH dropped” samples (red columns) represent pH 5.0 ± 0.1, 0.1 M NaNO_3_, 0.1-0.6 M NaCl.

**Figure 8:**
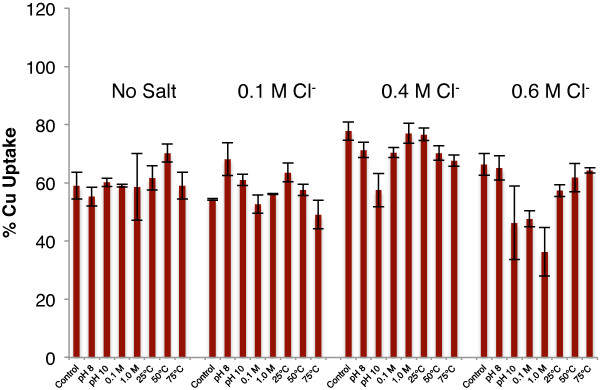
**Macroscopic % copper retention data for all particles at all salinities examined following exposure to NaCl for 24 hours at pH 5.0 ± 0.1.** Salinity increases from left to right as labeled.

Following the initial adsorption phase, 70-80% of the copper in solution was retained (Figure 7a-d). The subsequent addition of sodium chloride improves copper retention for all sets of aggregates. As the salinity was increased, the percent copper retained generally reached a maximum at 0.4 M chloride. Upon comparing only the “pH dropped” samples, the trend showing a retention maximum at 0.4 M Cl^−^ is more readily apparent (Figure 8). In contrast with the Zn(II) results, the differences in retention between the various aggregates increase as salinity rises, as shown by an increasing standard error between samples as the chloride concentration increases (Table 4). These results indicate a fundamentally different behavior of copper compared to the zinc experimental results: as chloride concentration increases, characteristics of the individual nanoparticle aggregates, such as morphological, structural, surface charge, and surface area differences have a noticeable effect on copper retention.

**Table 4:** Average percent Cu(II) uptake and standard errors for “pH dropped” samples

	**Average (% uptake)**	**Standard error**
**No salt**	60.4%	1.54%
**0.1 M Cl**^**−**^	57.8%	2.19%
**0.4 M Cl**^**−**^	71.1%	2.34%
**0.6 M Cl**^**−**^	55.7%	3.88%

#### Geochemical modeling

**Figure 9:**
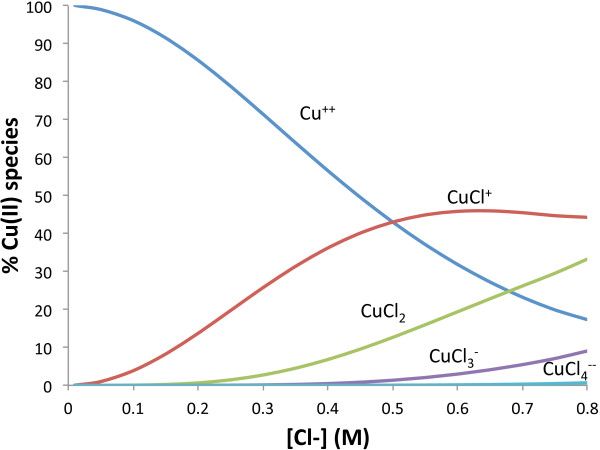
Speciation diagram of Cu(II) chloride species at a range of chloride concentrations and a Cu(II) concentration of 0.131 mM and pH 5.0.

While Cu^2+^ is the dominant aqueous copper species at low chloride concentrations, CuCl^+^ becomes the dominant aqueous complex at ~0.5 M chloride. In the macroscopic results, a retention maximum at 0.4 M chloride was evident before a decline in copper retention at 0.6 M chloride, which correlates with CuCl^+^ becoming dominant in solution at 0.5 M chloride and the significant formation of other copper-chloride complexes (CuCl_2_, CuCl_3_^−^, and CuCl_4_^2−^) (Figure 9). The presence of these stable complexes likely hinders further copper sorption, leading to the decline in copper retention beyond 0.4 M chloride.

#### Spectroscopic results

**Figure 10:**
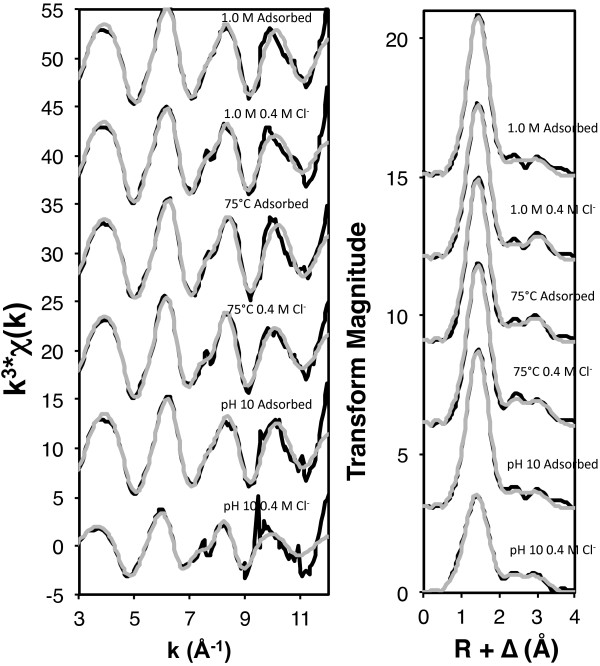
**Copper K-edge EXAFS spectra and Fourier transforms (black) with overlain fits (grey) for “adsorbed” and “pH dropped” samples (0.4 M Cl**^**− **^**) for particles aggregated at 1.0 M, 75°C, and pH 10.**

The Cu K-edge EXAFS spectra of the adsorbed and desorbed samples display visible differences (Figure 10) including a shoulder feature at k = ~7.5 Å^−1^ that is more pronounced in the spectra of the desorbed samples. The first-shell neighbor was best fit with Cu-O scattering interactions, with an average coordination number of 4.3 (range: 3.6-4.7) and an average interatomic distance of 1.96 Å (range: 1.95-1.97 Å) (Table 5) for all samples, consistent with the equatorial Cu-O bonds in a Jahn-Teller distorted octahedral Cu(II) complex [46,47]. These results correspond with previous spectroscopy studies of Cu(II) sorption onto mineral surfaces [46,48].

**Table 5:** **Results from K-edge EXAFS fitting of Cu samples (see Figure**10**for corresponding EXAFS spectra and Fourier transforms)**

		**Cu-O**		**Cu-Fe 1**		**Cu-Fe 2**		
**Aggregation condition**	**Sorption step**	**CN**	**R(Å)**	**CN**	**R(Å)**	**CN**	**R(Å)**	**r-factor**
**1.0 M**	**Adsorbed**	4.3 ± 0.9	1.96 ± 0.02	1.3 ± 0.6	2.96 ± 0.04	0.8 ± 0.6	3.44 ± 0.07	0.0058
**1.0 M**	**0.4 M Cl**^**−**^	4.7 ± 1.0	1.97 ± 0.02	1.4 ± 0.6	2.97 ± 0.04	1.3 ± 0.6	3.44 ± 0.05	0.0059
**75°C**	**Adsorbed**	4.4 ± 1.0	1.95 ± 0.02	1.4 ± 0.6	2.94 ± 0.04	1.3 ± 0.7	3.43 ± 0.05	0.0059
**75°C**	**0.4 M Cl**^**−**^	4.7 ± 1.0	1.97 ± 0.02	1.9 ± 0.6	2.99 ± 0.03	1.3 ± 0.6	3.48 ± 0.05	0.0052
**pH 10**	**Adsorbed**	4.5 ± 0.9	1.96 ± 0.02	1.3 ± 0.5	2.95 ± 0.03	0.9 ± 0.4	3.44 ± 0.06	0.0052
**pH 10**	**0.4 M Cl**^**−**^	3.6 ± 0.6	1.97 ± 0.01	1.5 ± 0.3	2.99 ± 0.02	0.9 ± 0.3	3.46 ± 0.04	0.0035

Included are coordination numbers (CN), interatomic distances (R), and goodness of fit values (R-factor). Debye-Waller values were allowed to float for first-shell fits (average: 0.004 Å^2^) and were fixed at 0.01 Å^2^ for subsequent shells.

During EXAFS fitting, the inclusion of a second shell Cu-Cl bond with a coordination number of 1 and bond length of approximately 2.2 Å [49] was attempted to explore the possibility of Cu-Cl sorption species. However, this caused Cu-O coordination numbers to decline to unrealistic numbers and provided unreasonable fits, so we have not included Cu-Cl interactions between the first Cu-O shell and the second Cu-Fe shell. The second shell was best fit with a Cu-Fe neighbor with an average coordination number of 1.5 (range: 1.3-1.9) and an average interatomic length of 2.97 Å (range: 2.94-2.99 Å); the third shell was also best fit with a Cu-Fe neighbor with an average coordination number of 1.3 (range: 0.8-1.3) and an average interatomic length of 3.44 Å (range: 3.43-3.48 Å) (Table 5). The shorter Cu-Fe distance (2.94-2.99 Å) has been associated with the formation of inner-sphere edge sharing complexes [50]. Similar bond lengths have been reported for Cu-Cu bonds corresponding to the formation of dimers [51], but our best fit was obtained with the inclusion of a Cu-Fe shell at this length. The longer Cu-Fe distance (3.43-3.48 Å) most accurately corresponds with the presence of mononuclear monodentate complexes, as modeled or interpreted by other investigators [52-54]. Visual changes in the EXAFS spectra therefore likely correspond to changes in the proportions of these species as a result of the pH-lowering desorption step. Based on the lack of chloride in the EXAFS fits, it is not likely that chloride plays a direct role in the sorption mechanism, e.g. through the formation of ternary surface complexes; however, it may play an indirect role by initially reducing positive surface charges (thereby improving copper retention) and then, at higher concentrations, by forming stable aqueous Cu-Cl complexes (reducing copper retention). Our copper speciation diagram indicates the formation of aqueous copper chloride species as chloride concentration increases, supporting the relationship between aqueous copper speciation and retention behavior (Figure 9).

## Conclusions

Based on these model studies, iron oxyhydroxide nanoparticles could be a useful tool for removing zinc from contaminated water supplies that lead to the ocean. Copper retention, in contrast, appears to be optimized at an intermediate salinity consistent with brackish water, and therefore may release considerable fractions of retained copper at higher (e.g. seawater) salinity levels; copper retention also appears to become more variable and dependent on aggregation mechanism at these increasing salinities. Accordingly, strategies for the environmental remediation of metal-contaminated waters should take into account potential changes in geochemical parameters that may induce aggregation, increase salinity, and affect solution pH.

## Competing interests

The authors declare that they have no competing interests.

## Authors’ contributions

RC ran the DLS and XRD analyses and macroscopic (atomic absorption spectroscopy) experiments, created the speciation diagrams, carried out the curve fitting of the EXAFS data, and drafted the manuscript. CK collected all EXAFS data and drafted the manuscript. All authors read and approved the final manuscript.
