# Erratum to: Zn (II) and Cu (II) adsorption and retention onto iron oxyhydroxide nanoparticles: effects of particle aggregation and salinity

**DOI:** 10.1186/s12932-015-0032-2

**Published:** 2015-11-19

**Authors:** Rebecca B. Chesne, Christopher S. Kim

**Affiliations:** School of Earth and Environmental Sciences, Schmid College of Science & Technology, Chapman University, Orange, CA 92866 USA

## Erratum to: Geochemical Transactions (2014) 15:6 DOI 10.1186/1467-4866-15-6

In the original version of this article errors in Figs. 5 and 9 were identified by the authors. The corrected figures are given below.

**Fig. 5:**
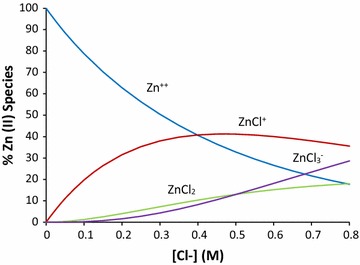
Speciation diagram of ZN(II) chloride species at a range of chloride concentrations and a Zn(II) concentration of 0.046 mM at pH 5.0

**Fig. 9:**
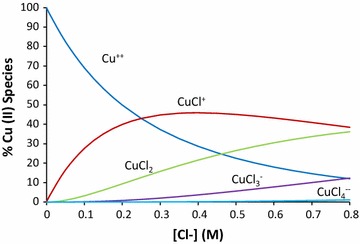
Speciation diagram of Cu(II) chloride species at a range of chloride concentrations and a Cu(II) concentration of 0.131 mM and pH 5.0

